# SARS-CoV-2 BW lineage, a fast-growing Omicron variant from southeast Mexico bearing relevant escape mutations

**DOI:** 10.1007/s15010-023-02034-7

**Published:** 2023-04-14

**Authors:** Rodrigo García-López, Xaira Rivera-Gutiérrez, Mauricio Rosales-Rivera, Selene Zárate, José Esteban Muñoz-Medina, Benjamin Roche, Alfredo Herrera-Estrella, Bruno Gómez-Gil, Alejandro Sanchez-Flores, Blanca Taboada, Carlos F. Arias

**Affiliations:** 1 Departamento de Genética del Desarrollo y Fisiología Molecular, Instituto de Biotecnología, Universidad Nacional Autónoma de México, Cuernavaca, Morelos Mexico; 2 Posgrado en Ciencias Genómicas, Universidad Autónoma de la Ciudad de México, Mexico City, Mexico; 3 Coordinación de Calidad de Insumos y Laboratorios Especializados, Instituto Mexicano del Seguro Social, Mexico City, Mexico; 4 MIVEGEC, Université de Montpellier, IRD, CNRS, Montpellier, France; 5 Departamento de Etología, Fauna Silvestre y Animales de Laboratorio, Facultad de Medicina Veterinaria y Zootecnia, Universidad Nacional Autónoma de México (UNAM), Mexico City, Mexico; 6 Laboratorio Nacional de Genómica Para La Biodiversidad-Unidad de Genómica Avanzada, Centro de Investigación y de Estudios Avanzados del IPN, Irapuato, Mexico; 7 Centro de Investigación en Alimentación y Desarrollo AC, Unidad Mazatlán, Mazatlán, Mexico; 8 Unidad Universitaria de Secuenciación Masiva y Bioinformática, Instituto de Biotecnología, Universidad Nacional Autónoma de México, Cuernavaca, Morelos Mexico

**Keywords:** Genomic surveillance, SARS-CoV-2 variants, COVID-19, Omicron, Mutations

## Abstract

**Purpose:**

The swift expansion of the BW.1 SARS-CoV-2 variant coincided with a rapid increase of COVID-19 cases occurring in Southeast Mexico in October, 2022, which marked the start of Mexico’s sixth epidemiological wave. In Yucatan, up to 92% (58 of 73) of weekly sequenced genomes between epidemiological week 42 and 47 were identified as either BW.1 or its descendant, BW.1.1 in the region, during the last trimester of 2022. In the current study, a comprehensive genomic comparison was carried out to characterize the evolutionary history of the BW lineage, identifying its origins and its most important mutations.

**Methods:**

An alignment of all the genomes of the BW lineage and its parental BA.5.6.2 variant was carried out to identify their mutations. A phylogenetic and ancestral sequence reconstruction analysis with geographical inference, as well as a longitudinal analysis of point mutations, were performed to trace back their origin and contrast them with key RBD mutations in variant BQ.1, one of the fastest-growing lineages to date.

**Results:**

Our ancestral reconstruction analysis portrayed Mexico as the most probable origin of the BW.1 and BW.1.1 variants. Two synonymous substitutions, T7666C and C14599T, support their Mexican origin, whereas other two mutations are specific to BW.1: S:N460K and ORF1a:V627I. Two additional substitutions and a deletion are found in its descending subvariant, BW.1.1. Mutations found in the receptor binding domain, S:K444T, S:L452R, S:N460K, and S:F486V in BW.1 have been reported to be relevant for immune escape and are also key mutations in the BQ.1 lineage.

**Conclusions:**

BW.1 appears to have arisen in the Yucatan Peninsula in Southeast Mexico sometime around July 2022 during the fifth COVID-19 wave. Its rapid growth may be in part explained by the relevant escape mutations also found in BQ.1.

**Supplementary Information:**

The online version contains supplementary material available at 10.1007/s15010-023-02034-7.

## Introduction

**Fig. 1:**
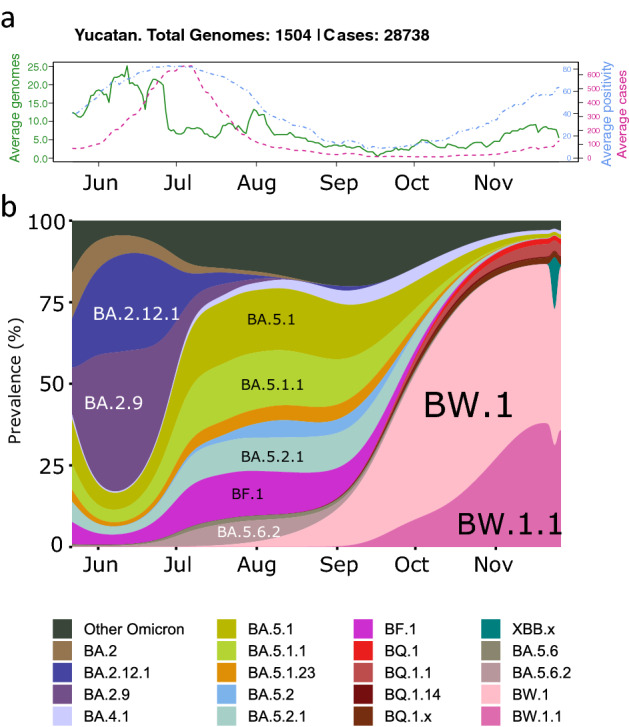
Epidemiological and genomic data in the State of Yucatan from May 22 to November 26, 2022. **a** Daily averages of confirmed cases (green solid line), positivity rate (dashed blue line), and total available genomes (dashed magenta line). Each line is presented with its own color coded y-axis. **b** Variant prevalence of SARS-CoV-2 variants. Variants accounting for less than 1% are collated into “Other Omicron”

As the COVID-19 pandemic approached its third year, most countries had undergone major changes in their fight against its etiological agent, coronavirus SARS-CoV-2 [1]. Stringent health care regulations had shifted to a reactive, rather than preemptive, set of measures, whilst major governmental surveillance efforts gave way to self-testing and restrictions were lifted. Consequently, as 2023 drew near, an unprecedented variability of Omicron-only subvariants dominated an ever-shifting global landscape [2, 3], which highlighted the utmost importance that genomic surveillance had played. The evolution of the SARS-CoV-2 genome has been described for more than three years by an unprecedented global genomic surveillance effort (14,941,263 sequences have been deposited into GISAID [4] by February 15, 2023). This worldwide cooperation towards open data has allowed scientists from all countries to contribute towards the characterization of major evolutionary paths that the virus has transited in response to natural and vaccine-acquired immunity [5]. Here, we present BW.1 (an alias for BA.5.6.2.1), an Omicron subvariant descending from the BA.5.6.2 lineage, that may have arisen in Mexico in early July and rapidly became dominant in the Yucatan Peninsula (Southeast) in October (Fig. 1), and its descendant, BW.1.1, which spread in late September and by late November had a similar prevalence to BW.1. The BW.1 variant carries significant immune escape mutations that are shared with those found in the BQ.1 variant, belonging to one of the most rapidly spreading lineages described to date (namely, mutations S:K444T, S:L452R, S:N460K, and S:F486V). Instead, BW.1.1 variant has the mutation S:F486A.

### Surveillance in Mexico

Within Latin America, Mexico has been one of the countries that have sequenced the most SARS-CoV-2 genomes (85,329 as of the time of writing), second only to Brazil (217,501). This has been in part thanks to an unprecedented collaborative sequencing effort carried out by the government (INMEGEN, InDRE) and multiple academic institutions (CoViGen-Mex). The resulting sequences have enabled the study of the variant turnover in the country throughout the three years of the pandemic [6, 7]. As pointed out in previous studies [8, 9], the Yucatan Peninsula (States of Campeche, Quintana Roo, and Yucatan) is one of the primary entry points for SARS-CoV-2 variants in Mexico due to its large influx of tourists and commercial activities in the areas surrounding Merida and Cancun. During late 2022, the Yucatan Peninsula underwent a period of high SARS-CoV-2 transmission, which may have probably been driven by the BW lineage (Supplementary Fig. 1), most prominently in the state of Yucatan. Coincidentally, starting in October 2022, the state registered a rapid increase in cases that foreshadowed the onset of a new epidemiological surge in the region (Fig. 1a) [10]. Other States in the country soon followed, and the BQ lineage became dominant in most areas except the Yucatan Peninsula (SE in Supplementary Fig. 1), possibly because the BW lineage had already occupied this niche. The positivity rate (the incidence of positive cases among all cases) in the state of Yucatan is presented in Fig. 1a as an additional estimator of active transmission, as confirmed cases dwindled after the previous wave. During the same period, as shown in Fig. 1b, the BW.1 variant managed to outcompete every other variant in the region, including its parental variant BA.5.6.2. An important limitation of the current study is that it encompasses an inter-wave period, which had the historically lowest number of total cases, resulting in a restricted number of available genomes, which impacts prevalence calculations. This pattern was observed both in Mexico and throughout the world.

### Origins of the BW lineage

**Fig. 2:**
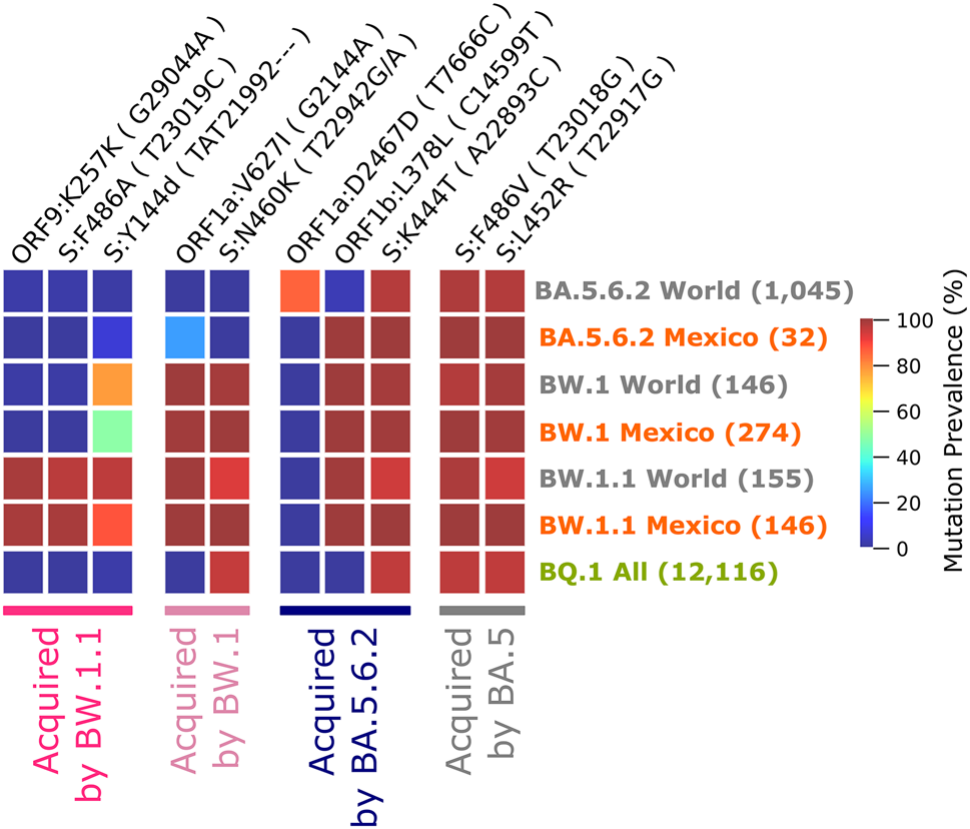
Prevalence of mutations acquired by BW variants and BA.5.6.2 and comparison with BQ.1. World sequences exclude Mexico. BQ.1 sequences include all available sequences from that variant

From a genomic perspective, all BA.5.6.2 sequences collected in Mexico bear two major differences from those from the rest of the world that match those in lineage BW: the addition of transition C14599T (ORF1b:L378L, present in all Mexican [M] BA.5.6.2 genomes but only in 3.06% in the rest of the world [W]) and the absence of transition T7666C (ORF1a:D2467D: M:0%, W:85.45%), as seen in Fig. 2. Most genomic sequences of BW.1 in the world differ from their parental BA.5.6.2 by two point mutations: transition G2144A (ORF1a:V627I) and transversion T22942G (S:N460K). By January, 2023, a subset of BW.1 sequences were reclassified as BW.1.1, which differs from their parental BW.1 variant in three additional changes: transition T23019C (S:F486A), synonymous transition G29044A (ORF9:K257K), and deletion TAT21992–- (S:Y144del), all of which were found in > 90% of all BW.1.1 genomes worldwide. Most importantly, S:Y144del was detected in 77.68% of BW.1 genomes from Mexico but only 47.81% in those from other countries. A detailed map of all the relevant mutations throughout time can be found in Supplementary Fig. 2.

The estimated relative growth advantage per week of BW.1 over BA.5.6.2 was 40% (95% CI 36–47%) for the study period. Similarly, BW.1.1 has an advantage of 28% (95% CI 16–41%) over BW.1 for the same period.

**Fig. 3:**
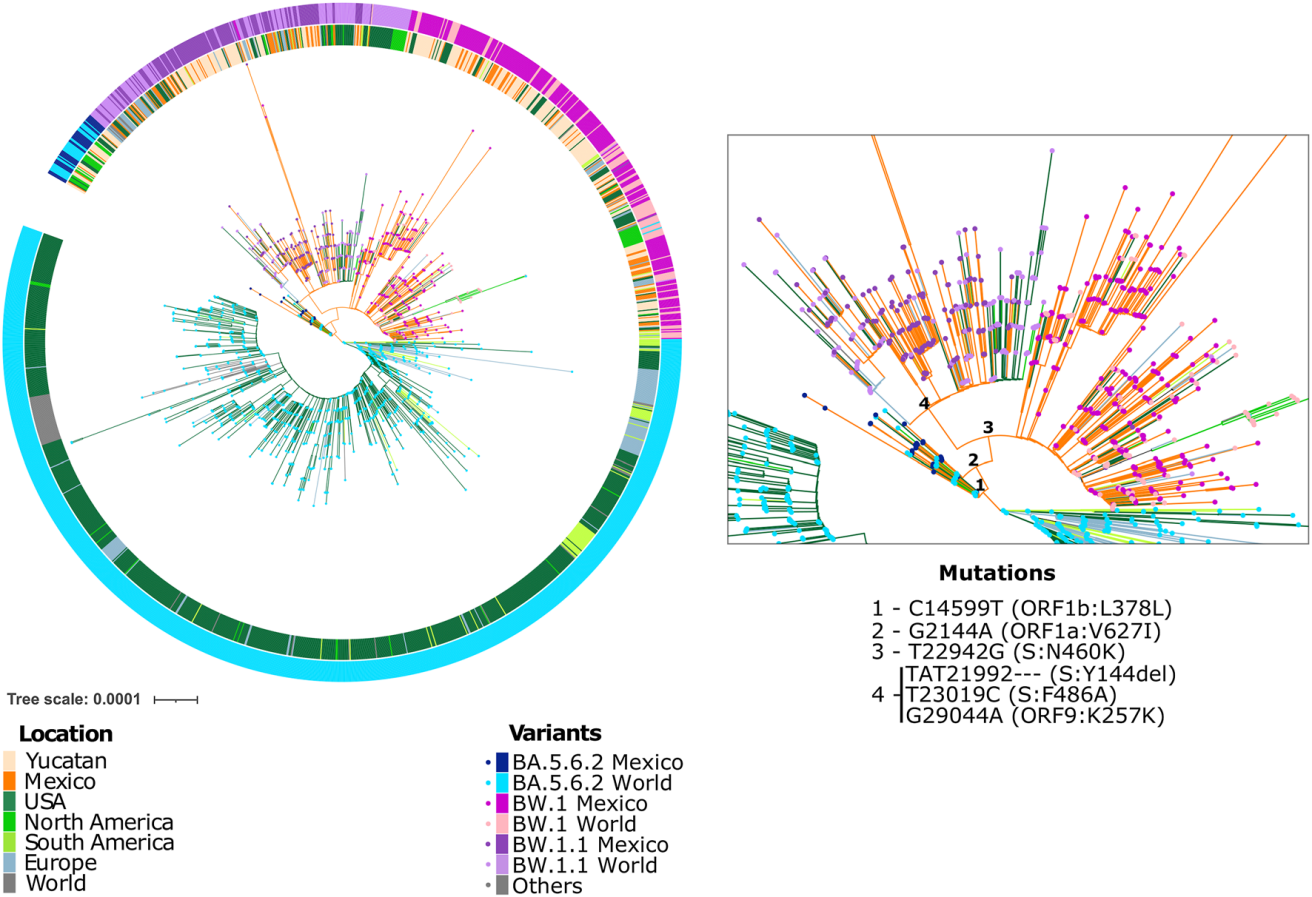
Phylogenetic reconstruction of BW linage and BA.5.6.2 genomes collected in Mexico and the rest of the world with ancestral sequence reconstruction and coloured according to the maximum likelihood estimation of ancestral country of origin. The figure shows a subtree from the complete reconstruction, clipped at a node defined by mutation C8605T (midpoint root), a key mutation in BA.5.6.2 and descendants. Each bullet represents a genome and each color a variant. The outer circle indicates the assigned lineage for each sequence and matches variant color. The inner circle shows the sampling location of each virus. Branches are coloured according to the country of origin with the highest likelihood (> 0.99) for ancestral states. North America stands for Canada and Puerto Rico. Mexico includes other states besides Yucatan. The zoom on the right shows the mutational events associated with each ancestral state of the BW lineage. Numbers 1–3 show BW.1 key mutations. Number 4 shows three key mutations in BW.1.1

To investigate the evolutionary history of the BW lineage, a reconstruction of ancestral sequences (full phylogeny shown in Supplementary Fig. 3), and a reconstruction of ancestral regions were carried out to investigate the geographical origin of this lineage (Fig. 3). These analyses fully supported the hypothesis that BW.1 was originated in Mexico (> 0.99 marginal probability) during the fifth epidemiological wave (June to September 2022), as it derived from BA.5.6.2 viruses circulating in the state of Yucatan bearing substitution C14599T (marked with number 1 in Fig. 3). BW.1-specific mutations, S:F486A and S:N460K, were acquired subsequently (numbers 2 and 3, respectively in Fig. 3) and all BW.1 sequences are then placed in a monophyletic clade. The BW.1.1 subclade may have also originated in Mexico (> 0.99 probability), derived from the acquisition of the three additional changes described before (number 4 in Fig. 3).

### The BW lineage was most prevalent in Mexico

By November 5, 2022, nearly one month after the onset of the sixth wave in the Yucatan Peninsula, 134 out of 161 (83.22%) reported BW.1 genomes in GISAID had originated from Mexico (Supplementary Fig. 4) [4]. By November 26, 2022, BW.1 may have already been exported (274 of 386 genomes from Mexico, the rest distributed among 18 countries). Additionally, the earliest BW.1 sequences were collected in Mexico during epidemiological week 31 (early August) (Supplementary Fig. 4). In contrast, the earliest samples from the rest of the world were collected between weeks 37 and 39 (September in the Netherlands and the UK, respectively). In Mexico, most samples of BW.1’s parental variant, BA.5.6.2, were collected in the state of Yucatan (83.33% [*n* = 20]; Fig. 1). Additionally, in Mexico, the first gradual increase of BW.1.1 was observed beginning in week 38 (late September; Supplementary Fig. 4). In the rest of the world, this occurred in week 41 (early October).

### Important mutations found in lineage BW

Interestingly, the Yucatan Peninsula was the only region in Mexico where variants from the BQ lineage (derived from Omicron BA.5.3) failed to reach a clear dominance during the sixth wave (Supplementary Fig. 1). This is likely due to the fact that BW variants had already spread in this region and cases peaked weeks earlier [10]. In the State of Yucatan, the BW lineage was most likely responsible for the early onset of the sixth wave, whereas in other parts of the country, cases increased later and were dominated by BQ lineages. The BW lineage descends from Omicron lineage BA.5.6 and shares some mutations with BQ.x variants as they both descend from the BA.5 lineage (Fig. 2). From this parental lineage, BQ and BW inherited over 54 changes, including key mutations S:L452R (known to confer spike stability, viral fusogenicity, and increased infectivity [11]) and S:F486V (shown to decrease the effectivity of multiple monoclonal antibodies [12]), in the receptor binding domain (RBD) of the spike protein (S), a crucial region for antibody recognition [13].

Since the second half of 2022, immune selection has seemingly become one of the major driving forces behind the fixation of additional key genomic mutations through parallel and convergent evolution, particularly of those in the RBD. Due to the pressure of the incremental immunity in the population, both the BQ.1 and BW.1 variants have independently fixed two additional key RBD mutations: S:K444T and S:N460K (arising from different nucleotide changes). It has been reported that mutation S:K444T enhances viral resistance to bebtelovimab and P2G3 in Delta and hinders antibody recognition in Omicron BA.4 subvariants [14], as well as neutralization resistance, and evasion of Class 3 antibody recognition in BQ.1 and BQ.1.1 [15]. Mutation S:N460K, has been suggested as a driver for enhanced fusogenicity [15], syncytia formation, enhancement of S processing in S1 and S2 subunits on BA.4 and BA.5 Omicron variants, as well as enhanced neutralization resistance with subvariants BQ.1, BQ.1.1, BA.2 and BA.2.75 [15, 16]. Immune escape has been shown to be more efficient whenever S:K444T and S:N460K co-occur, as in BQ.1 and BW.1, impairing the effectiveness of monoclonal antibodies Evusheld [3] and bebtelovimab [14].

Of note, other phylogenetically distant lineages have also independently acquired these two mutations, such as the highly transmissible recombinant XBB, which is derived from BA.2.75.3 and BA.2.10.1 variants, whose genomes include the mutation S:N460K. Similarly, multiple phylogenetically distant subvariants of the BA.2 (such as BR.4 and CH.1), BA.4 (BA.4.6.3, CS.1), and BA.5 (CK.1, DB.1) clades have acquired different substitutions, resulting in variations in the S:K444 amino acid (in the case of BA.5.6.2 and descendants, S:K444T). Ultimately, predicting the dynamics of future outbreaks remains nearly impossible as it depends on very specific regional conditions. However, genomic surveillance allows for preemptively tracking important mutations which may contribute towards enhanced viral phenotypes that might pose an actual threat to human health, as the current study suggests for BW.1.

## Conclusions

The coordinated effort on Mexican genomic surveillance of SARS-CoV-2 has identified and characterized variants in the BW lineage bearing four prominent RBD key mutations S:L452R, S:F486V, S:K444T, and S:N460K. These are the same mutations that provide BQ.1 and its descendants with enhanced infectious and immune evasion capabilities. BW.1 appears to have originated from Mexican BA.5.6.2 variants in Yucatan (a buoyant international tourist destination), as these parental variants had already diverged from BA.5.6.2 variants in the rest of the world and inherited their genomic composition vertically to BW.1 and BW.1.1. The incorporation of mutations S:N460K and ORF1a:V627I kickstarted BW.1 into a distinct clade, which has since been detected in other countries but has not become widespread. In Mexico, BW.1 emerged during the previous epidemiological surge and has since become a major player in the Yucatan Peninsula, where BW.1 and BW.1.1 cases may have dominated during the sixth wave. BQ variants, which predominated elsewhere in Mexico, failed to spread in this region, possibly because they share a similar set of mutations and cannot outcompete one another. Future studies will tell if the advance of both lineages might be halted by the arrival of variants in the XBB lineage that have become the major driving force of the pandemic in early 2023.

## Methods

### Data collection and sequence alignments

From the period spanning weeks 21 to 47 (May to November 2022), all SARS-CoV-2 genomes from variants BQ.1 (12,367), BA.5.6.2 (1,101), BW.1 (388), and BW.1.1 (308) available on February 15, 2023 were downloaded with their associated metadata from GISAID [4], including lineage information based on PANGO v.4.2.0[17]. The ending day was selected as there was a large gap in genome sequencing in Yucatan starting in December. Nucleotide contents were analyzed in the 14,164 genomes, and those containing 10% or more ambiguous sequences (Ns) were removed from the study. Sequences from samples with no identifiable collection date were also ignored. Filtered sequences (BA.5.6.2 = 1,077, BQ.1 = 12,118, BW.1 = 386, and BW.1.1 = 301) were then aligned with Nextclade’s Nextalign default procedure (part of the Nextstrain pipeline) v2.11.0 and v.2.3.0 [18] using Wuhan-Hu-1 (NC_045512.2) as the reference genome, and BQ.1 variants were extracted from the alignment. Next, UShER v.0.6.2 [19] was used to retrieve relevant reference sequences (~ 2000 sequences were selected out of 14,028,940 on February, 17), and these were downloaded from NCBI and GISAID on the same date and appended to our dataset (a full list of IDs is reported in Supplementary Table 1). These were aligned again using Nextclade’s Nextalign with default parameters and a maximum parsimony reconstruction was carried out, using iqtree v2.1.2 [20] with the GTR + R4 + F evolutionary model. The resulting tree was used for an ancestral state reconstruction with TreeTime v0.9.5 [21] by parsimony, using default parameters (the resulting tree is shown as Supplementary Fig. 3), and then clipped at a node defined by mutation C8605T (midpoint root). Metadata on the sampling location were used to determine the putative origin of the BW.1 clade with a maximum likelihood ancestral region reconstruction made with PastML v1.9.34 [22] using the Marginal Posterior Probabilities Approximation (MPPA) method. Tree visualization and edition were carried out with iTOL [23] (the resulting tree is included in Fig. 3).

Using the MSA, in-house R scripts (see Data Availability) were used to identify and compare mutations by their nucleotide position in relation to longitudinal data and lineage, obtaining the prevalence in each subgroup of genomes (weeks/lineage) using R programming language R.4.2.2 [24]. The mutation prevalence at each week was summarized in a heatmap constructed with clustermap (seaborn v.0.11.2 [25]; matplotlib 3.5.2 [26]) in Python 3 [27].

The estimated relative growth advantage was calculated according to the calculation by Chen, et al., 2021 [28] from covSPECTRUM for the study period. GISAID [4] was used to identify prevalence of particular mutations in the different SARS-CoV-2 lineages.

## Supplementary Information

Below is the link to the electronic supplementary material.

Supplementary file1 (PDF 4220 KB)

Supplementary file2 (XLSX 993 KB)

## Data Availability

Virus genome IDs and GISAID identification numbers or INSD Accession Numbers (GenBank) for the target sequences and the references are provided in the Supplementary Table 1, respectively. A full acknowledgment list can be consulted in GISAID EPI_SET_230222ws (https://doi.org/10.55876/gis8.230222ws). To foster reproducibility, a copy of the scripts used for the analyses is publicly available on GitHub at https://github.com/GAL-Repository/SARS-CoV-2_BW_lineage
