# Supplementary material for: SARS-CoV-2 BW lineage, a fast-growing Omicron variant from southeast Mexico bearing relevant escape mutations
Source: Infection. 2023 Apr 14;51(5):1549–55. doi: 10.1007/s15010-023-02034-7 (PMC10103656; doi:10.1007/s15010-023-02034-7)
Supplement: Supplementary file 1 — Supplementary file1 (PDF 4220 KB) [file 15010_2023_2034_MOESM1_ESM.pdf]

Title: SARS-CoV-2 BW lineage, a fast-growing Omicron variants from southeast Mexico bearing clinically relevant escape mutations

Journal name:Infection

Authors:Rodrigo García-López, Xaira Rivera-Gutiérrez, Mauricio Rosales-Rivera, Blanca Taboada, Selene Zárate, José Esteban Muñoz-Medina, Benjamin Roche, Alfredo Herrera- Estrella, Bruno Gómez-Gil, Alejandro Sánchez-Flores, Carlos F. Arias.

Correspondence: Rodrigo García-López (rodrigo.garcia@ibt.unam.mx) and Blanca Taboada (blanca.taboada@ibt.unam.mx) from Departamento de Genética del Desarrollo y Fisiología Molecular, Instituto de Biotecnología, Universidad Nacional Autónoma de México, Cuernavaca 62210, Morelos, Mexico

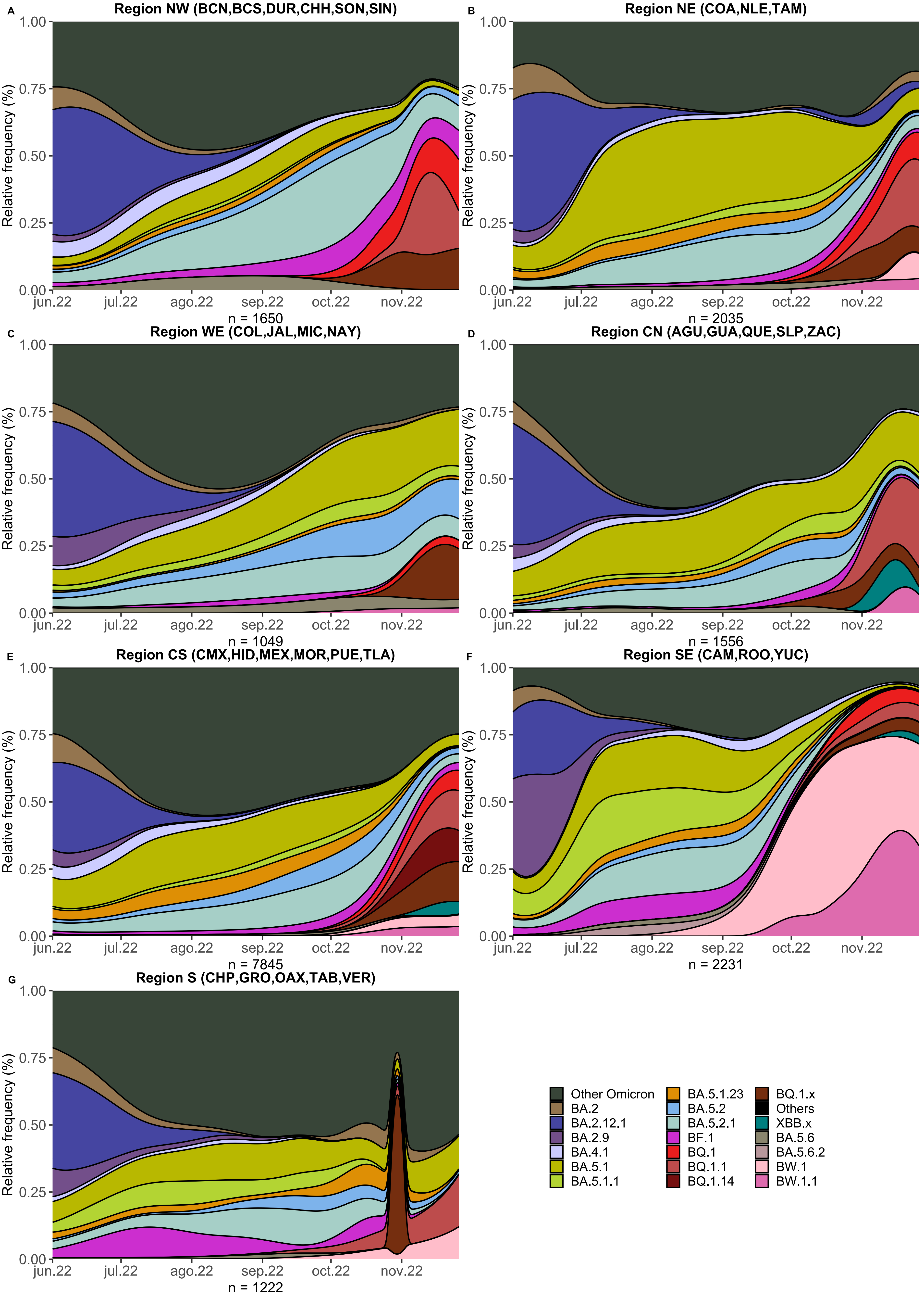

Supplementary Figure 1. Prevalence of SARS-CoV-2 variants in seven geographical regions of Mexico. The x axis shows weeks. Variants accounting for less than 1% are collated into "Other Omicron".

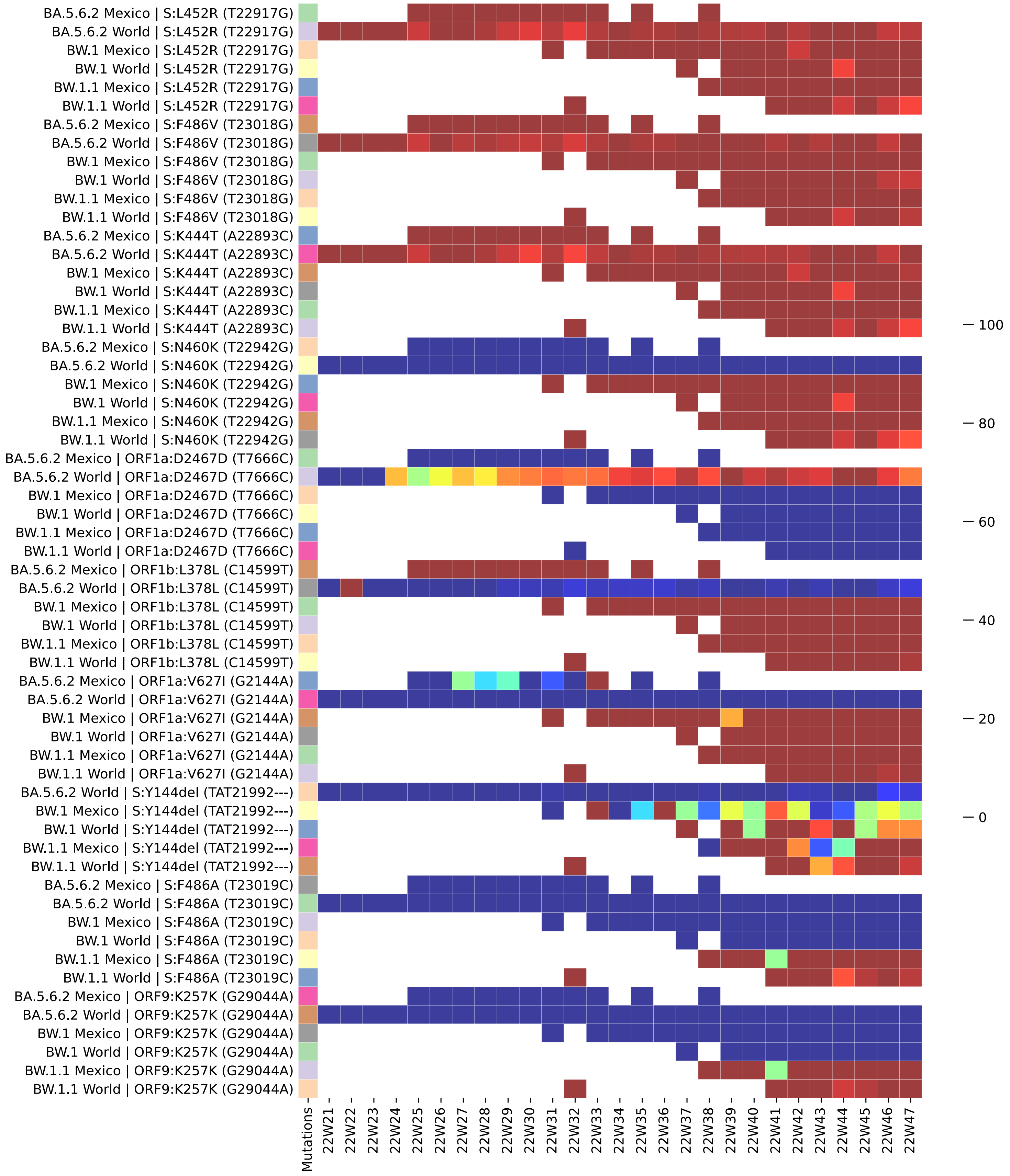

Supplementary Figure 2. Heatmap showing the longitudinal evolution of key mutations in genomes from Mexico and the rest of the world (y axis) from variants BA.5.6.2, BW.1 and BW.1.1 per week (x axis). Blank spaces represent an absence of samples

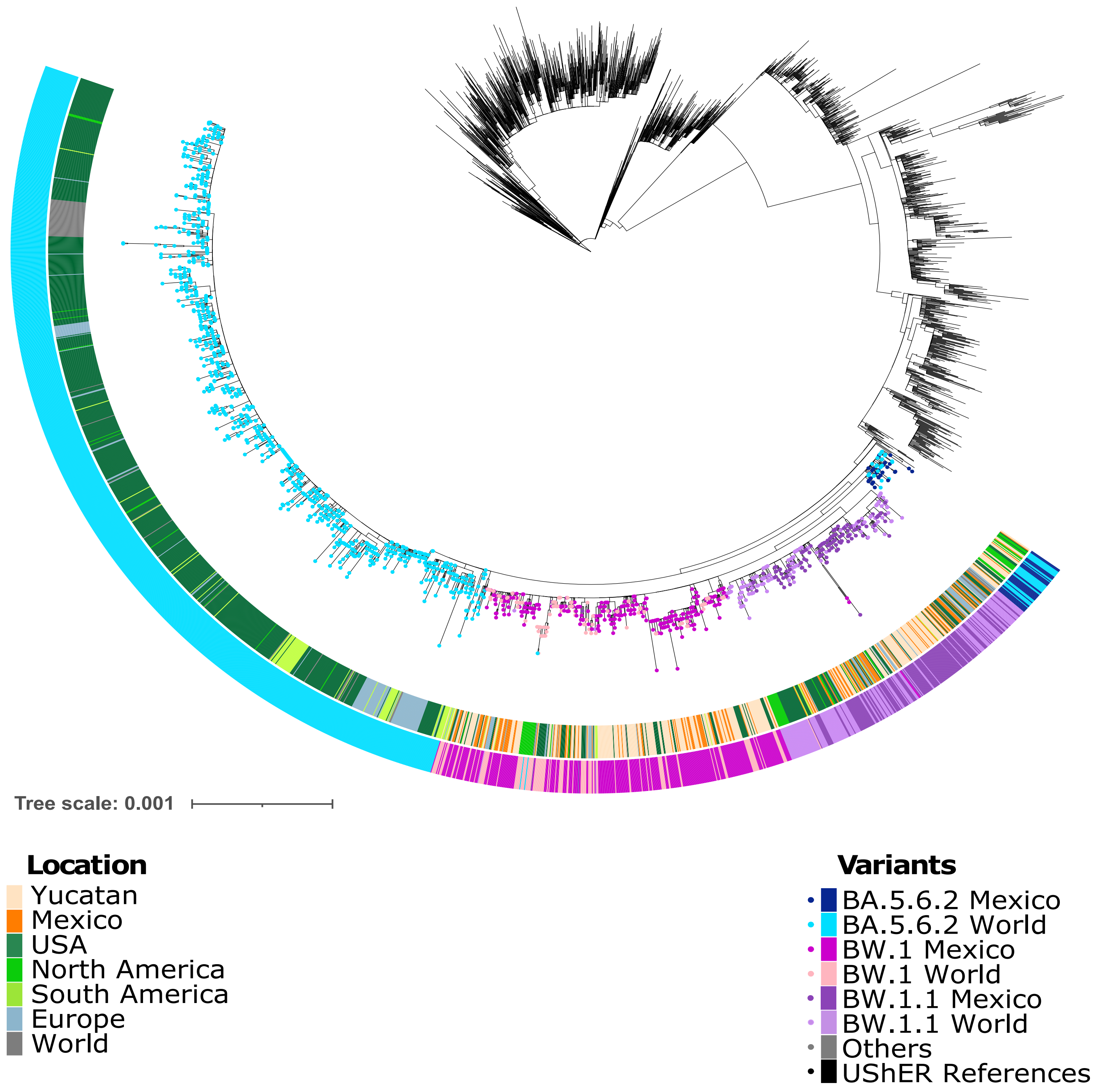

Supplementary Figure 3. Phylogenetic reconstruction of BW.x and BA.5.6.2 genomes wordwide, with reference sequences. Inner nodes show the ancestral state reconstruction of specific mutation events. Reference genomes were obtained using UShER. Each outer node represents a genome and each color a variant. The outer arch aids visualization of variants in the tree. The inner arch shows the collection location of each sample. North America stands for Canada and Puerto Rico. Mexico includes other states besides Yucatan.

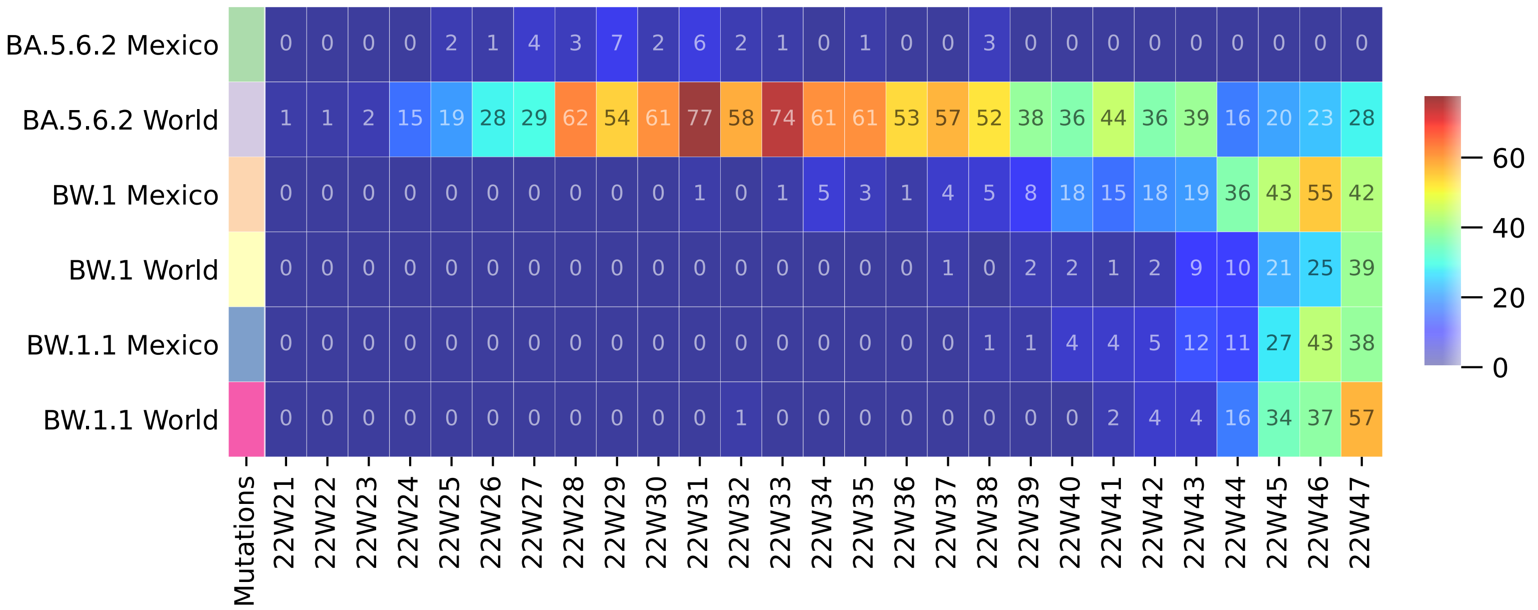

Supplementary Figure 4. Heatmap showing the weekly (x axis) incidence of SARS-CoV-2 variants BA.5.6.2, BW.1 and BW.1.1 in Mexico and the rest of the world (y axis). Numbers on each cell represent the absolute number of sequences in that week.
